# Generalized EmbedSOM on quadtree-structured self-organizing maps

**DOI:** 10.12688/f1000research.21642.2

**Published:** 2020-05-19

**Authors:** Miroslav Kratochvíl, Abhishek Koladiya, Jiří Vondrášek

**Affiliations:** 1 Institute of Organic Chemistry and Biochemistry of the CAS, Prague, Czech Republic; 2 Department of Software Engineering, Faculty of Mathematics and Physics, Charles University, Prague, Czech Republic; 3 Institute of Hematology and Blood Transfusion, Prague, Czech Republic

**Keywords:** dimensionality reduction, self-organizing maps, single-cell cytometry

## Abstract

EmbedSOM is a simple and fast dimensionality reduction algorithm, originally developed for its applications in single-cell cytometry data analysis. We present an updated version of EmbedSOM, viewed as an algorithm for landmark-directed embedding enrichment, and demonstrate that it works well even with manifold-learning techniques other than the self-organizing maps. Using this generalization, we introduce an inwards-growing variant of self-organizing maps that is designed to mitigate some earlier identified deficiencies of EmbedSOM output. Finally, we measure the performance of the generalized EmbedSOM, compare several variants of the algorithm that utilize different landmark-generating functions, and showcase the functionality on single-cell cytometry datasets from recent studies.

## Introduction

EmbedSOM is a dimensionality reduction (DR) algorithm for single-cell cytometry data, designed for high scalability, computational efficiency and performance ^1^. The design is based off FlowSOM ^2^, which utilizes unsupervised manifold learning by self-organizing maps (SOMs) to find structure in the high-dimensional data, and process the result into a meaningful and easily interpretable clustering of the dataset. So far, FlowSOM and SOMs in general seem to be the manifold learning and clustering method of choice for all kinds of cytometry based on protein-targeting antibodies, surpassing other clustering methods in precision, speed and scalability ^3^. EmbedSOM utilizes the same manifold learning method to extract information about the topology of an approximate manifold that describes the high-dimensional cell expression space, and uses it to quickly compute low-dimensional image of the cells that is suitable for visualization.

In this work, we focus on fixing inconsistencies and problems of the first version of EmbedSOM: First, we describe an updated version of EmbedSOM that improves the approximation to achieve mathematical smoothness of the projection. The brief description of EmbedSOM provided in the original paper is supplemented here by fully commented pseudocode, in order to aid scrutinization and interpretation of the method. Second, we review EmbedSOM as a generalized function for enriching a projection of selected landmarks to a projection of entire spaces. We demonstrate this by replacing the original SOMs with less-demanding t-SNE on random landmarks. Additionally, we describe GQTSOM, a novel variant of growing self-organizing maps (GSOMs, described e.g. by Rauber *et al.* ^4^) that was designed to alleviate precision and overcrowding problems of the original EmbedSOM. GQTSOMs utilize quad-tree space-partitioning structure to grow inwards, thus allowing the training algorithm to increase the resolution of manifold approximation on demand, and to benefit from the performance gain in early stages of training that is common to all GSOMs.

The functionality of the new algorithm is showcased on datasets that were recently used for studying other DR techniques. We show the differences between individual variants of landmark-generating functions, and provide visualizations comparable to those produced by current state-of-art algorithms. Finally, we demonstrate how the dynamic resolution of GQTSOMs aids detection of various small cell populations and rare cell types.

## Methods

### Landmark-directed embedding

**Figure 1.**
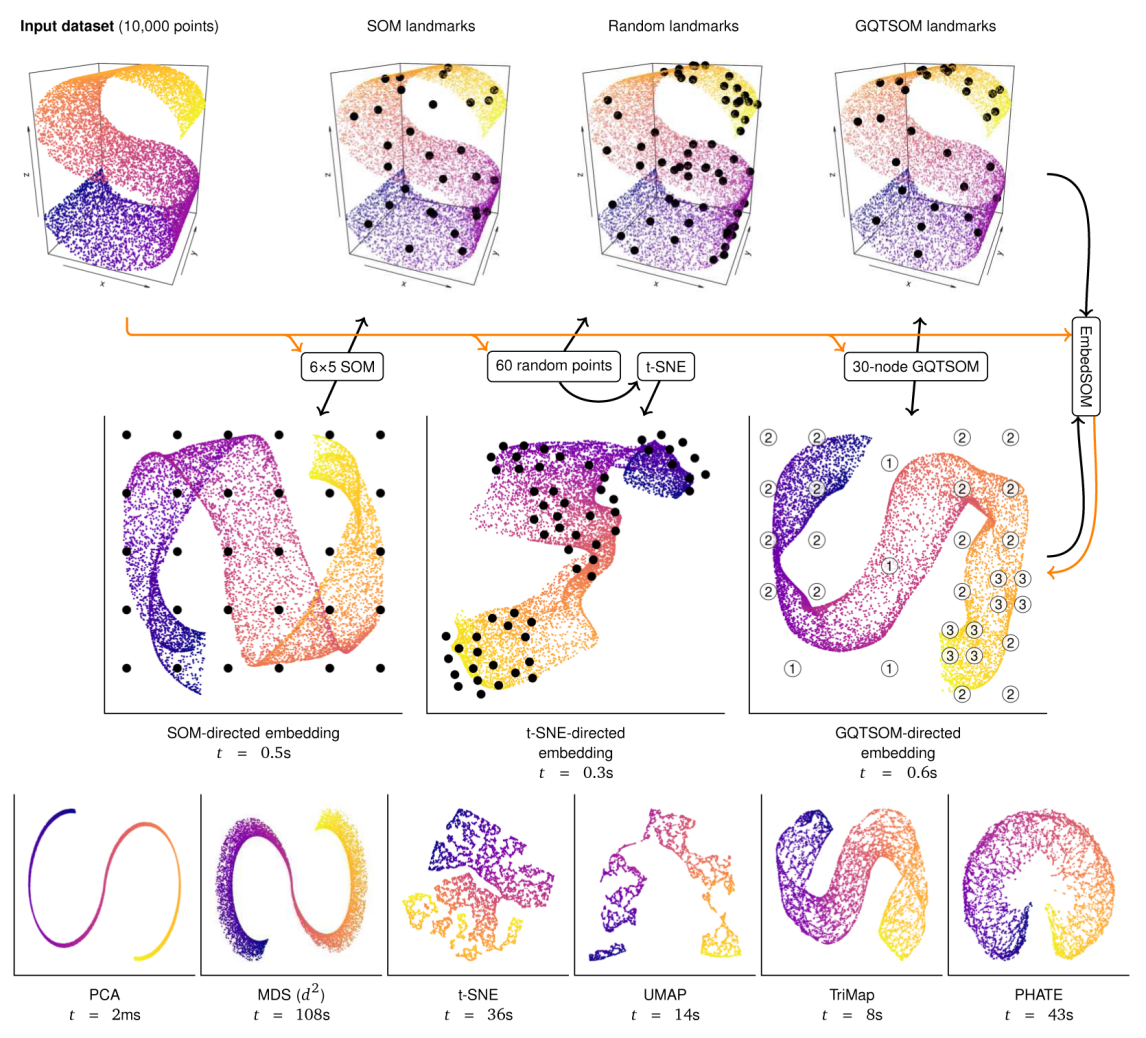
Overview of EmbedSOM interaction with landmarks on a toy dataset. Embedding process starts by reducing the input dataset (data flow is visualized as orange arrows) to landmarks (black arrows and dots) in high-dimensional (top row) and low-dimensional space (middle row). EmbedSOM quickly places the relatively large amount of individual input points into matching neighborhoods of the low-dimensional landmarks. The landmark-generating methods from left: A simple grid from SOM algorithm, a random selection of input points with 2-D topology reconstructed by t-SNE, and a GQTSOM-based grid. GQTSOM landmarks are labeled by their level in the quadtree. Visualizations from other methods ^5– 8^ (bottom row) are presented with computation time ( *t*) for comparison. R code that produces the plots is available in Supplementary material.

EmbedSOM projection can be viewed as an embedding enrichment method: From a set of landmarks in the high-dimensional space and a set of corresponding landmarks in the low-dimensional space, it produces a smooth function that maps all points from the higher-dimensional space to the low-dimensional space and preserves the relative neighborhoods of the landmarks. EmbedSOM was originally designed to work with simple SOM-originating landmarks, as shown in Figure 1.

We will refer to the high- and low-dimensional landmarks as *L* ϵ ℝ ^*n*× *D*^ and *l* ϵ ℝ ^*n*×2^. EmbedSOM embedding of a single high-dimensional point is achieved by reducing it to a collection of coordinates of its projections into subspaces that are generated by affine combinations of landmark pairs from *L*, and reconstructing it in low-dimensional space by reversing the process with corresponding landmark pairs from *l*.

The procedure is detailed as Algorithm 1. First, the algorithm chooses *k* landmarks closest to *X*, which are expected to give sufficient approximation. In lines 2–6 it computes scores for the *k* landmarks. The affine projection of *X* to a space defined by a pair of landmarks from *L* is computed at line 12 as *d*, its value is used to create a linear equation which has solutions at positions that would project to the same position *d* in the affine space generated from corresponding landmarks in *l*. After adding all parts of the approximation together, the linear system stored in *M* is very unlikely to remain singular. The position of embedded point is then obtained by simply solving the linear equation of 2 variables. Alternatively, one can view the algorithm as a minimization of the total squared error in all projected *d*:

$$\underset{x\epsilon {\mathbb{R}}^{2}}{\arg\min}\sum_{i,j}s_{i,j}{\left(d_{i,j}–\frac{\langle x–l_{i},l_{j}–l_{i}\rangle }{\langle \langle l_{j}–l_{i}\rangle \rangle }\right)}^{2}$$

Algorithm 1. **EmbedSOM projection from *D*-dimensional Euclidean space to 2-D using *n* landmarks.**

  1: **procedure** E mbedSOM( *X* ϵ ℝ ^*n*^, *L* ϵ ℝ ^*n*× *D*^, *l* ϵ ℝ ^*n*×2^, *k* ϵ {4 . . . *n*}, *m* > 0, *a* > 0)

  2: *c* ← a sequence of *c _i_* = 〈〈 *X* − *L _i_*〉〉 for *i* ϵ {1 . . . *n*}

  3: *o* ← indexes of *k* smallest elements of *c* in order

  4: *μ* ← $\sum_{i\leq k}\frac{c_{o(i)}}{i\cdot k}$                                                               ▷ estimate the distribution of landmark distances

  5: *σ* ← $\sqrt{\sum_{i\leq k}{\left(\frac{c_{o(i)}–\mu }{i}\right)}^{2}}$

  6: *S* ← a sequence of $S_{i}=\exp\frac{b\cdot \left(\mu –c_{o(i)}\right)}{\sigma }\cdot \left(1–\exp\frac{c_{o(i)}–c_{o(k)}}{m\cdot c_{o(k)}}\right)$ for *i* ϵ {1 . . . *k*}        ▷ compute scores

  7: $M\leftarrow \left(\begin{array}{cc} 0 & 0\\ 0 & 0 \end{array}\;|\begin{array}{c} \;0\\ \;0 \end{array}\right)$                                                                  ▷ accumulator for the linear equation system

  8: **for** *i′* ϵ {1 . . . *k* − 2} **do**                                                  ▷ iterate through pairs of *k* − 1 closest landmarks

  9: **for** *j′* ϵ { *i* + 1 . . . *k* − 1} **do**

10: *i* ← *o*( *i*′)                                                                            ▷ obtain non-permuted landmark indexes

11: *j* ← *o*( *j*′)

12: $d\leftarrow \frac{\langle X–L_{i}\text{,}L_{j}–L_{i}\rangle }{\langle \langle L_{j}–L_{i}\rangle \rangle }$                                                                 ▷ projection position in the affine space

13: *h* ← *l _j_* − *l _i_*                                                                                                                ▷ helper values

14: $y\leftarrow d+\frac{\langle h,l_{i}\rangle }{\langle \langle h\rangle \rangle }$

15: $s\leftarrow {\text{(}1+\langle \langle h\text{〉〉}\text{)}}^{–a}\cdot e^{{\left(d–\frac{1}{2}\right)}^{2}}\cdot S_{i^{\prime }}\cdot S_{j^{\prime }}\text{,}$                                    ▷ score for this pair of landmarks

16: $M\leftarrow M+s\;\cdot \;\left(\frac{h\cdot h^{T}}{\langle \langle h\rangle \rangle }\;|\;\begin{array}{c} y\cdot h_{1}\\ y\cdot h_{2} \end{array}\right)$                                   ▷ add this approximation to the linear system

17: **end for**

18: **end for**

19: **return** solution ( *x*_1_, *x*_2_ ) of the linear system in *M*

20: **end procedure**

Since the squared term is linear in *x*, the inner function is a quadratic form that can be minimized algebraically by finding zero of its derivation. This procedure gives the formulas used in the algorithm.

The algorithm can be easily expanded to embedding into general *P*-dimensional spaces by taking the low-dimensional landmarks *l* from ℝ ^*n*× *P*^, increasing the size of the matrix *M* for a linear equation of *P* variables, and solving a larger linear system at the end.

Notably, the initial reduction of the input data to one-dimensional projections to affine spaces ( *d* in the algorithm) prevents various complications from fitting the high-dimensional *distances* into low-dimensional space, avoiding many problems that arise from dimensionality overhead in other DR algorithms. Similar approach has been taken e.g. by TriMap ^5^, where the transferred information is reduced to mere binary relations between point distances.

**Embedding parameters** The embedding procedure admits several tunable parameters: *k* is the number of nearest landmarks used for the approximation, *m* > 0 is an arbitrary parameter that selects the steepness of score decay for distance order approaching *k*, *b* > 0 chooses the steepness of score decay for landmarks far from *X*, and *a* lowers the score of approximations to pairs of relatively far low-dimensional landmarks.

Parameter *m* is specifically designed to lower the score of landmarks with distances that approach *k*-closest landmark. As a result, small changes in the input point *X* can not cause sharp changes in the scores assigned to individual parts of the approximation. Consequently, EmbedSOM function is smooth in *X*.

Values of parameters *k*, *m*, and *a* must be chosen to avoid singularities and near-singularities when computing the final approximation, which may happen if the set of *s*_*i*, *j*_ contains insufficient number of higher-than-negligible scores. That may be caused mainly by setting too low values of *k* or *m*, or too high value of *b*. Argument setting of $k≃\sqrt{\left|L\right|}$, *m* = 10, *b* = *e* ^−1^ and *a* = 1 worked well in a majority of tested use cases and can be considered a good default.

**Embedding complexity** To compute a *P*-dimensional projection of a single point from a *D*-dimensional space, EmbedSOM projection conducts the following operations: | *L*| measurements of distances in high-dimensional space, sorting the *k* smallest elements of the distance vector of size | *L*|, and conversion of *k* distances to scores. On the landmark pairs, it conducts at most *k* ^2^ computations of scores *s*, the same number of computations of *d*_*i*, *j*_ from 2 dot-products in high-dimensional space, and computation of a partial *P*-by-( *P* + 1) matrix for solving the linear system in ℝ ^*P*^. Finally, the linear system is solved using Cramer’s rule. The total of computation times is thus, in respective order,

$$\left(D\cdot \left|L\right|\right)\;+\;\left(\log\;k\cdot \left|L\right|\right)\;+\;k^{2}\;\left(\left(1\right)\;+\left(D\right)+\left(P^{2}\right)\right)\;+\;\left(P!\right).$$

Assuming the default parameter setting and *P* ϵ {2, 3}, this complexity sums to ( *D* · | *L*|). The procedure can be trivially repeated for any number of input points.

**Different distance measures** We have assumed that the metric used in both high-dimensional and low-dimensional spaces is Euclidean. Generally, EmbedSOM behaves well even if the distance measure used for the scoring function is swapped for any function that acts as a metric on vector spaces, including the popular *L* ^1^ and *L* ^∞^ metrics.

Nevertheless, the computation of ‘projections’ using dot-products may then be viewed as a rather questionable reinterpretation of the point coordinates in an inner product space. Fortunately, the minimal-distance projection to a fixed subspace is a linear operator under both *L* ^1^ and *L* ^∞ 9^, which is sufficient for EmbedSOM computation even without requiring the inner product property.

**Relation to other dimensionality-reduction algorithms** The currently used non-linear dimensionality reduction methods are most often constructed from optimization tasks that optimize the embedding of the data points into the low-dimensional space, attempting to preserve selected properties from the high-dimensional space. The methods include t-SNE (optimizes Kullback-Leibler divergence between transformed distances in high-dimensional *k*-neighborhoods ^5^), UMAP (optimizes the cross-entropy between topological representations of the data ^6^), TriMap (optimizes the preservation of distance ordering in triplets of data points ^7^, MDS (optimizes the mean squared error between dissimilarity and distance matrices), isomap (optimizes walk-like distances on k-neighborhood graph used as dissimilarities in MDS), PHATE (uses a dissimilarity based on heat transfer potential in MDS ^8^), Kamada-Kawaii algorithm (uses simulation to optimize a spring model of a graph) and many others. Performance of such methods is most impacted by the necessity to examine a large subset of the $\left(\begin{array}{l} n\\ 2 \end{array}\right)$ relations between the *n* input data points.

This computationally expensive optimization can be traded off by first creating a smaller model of the data, and using it to find approximate embedding of the data points. This is used e.g. by scvis, which trains an autoencoder to represent the data by 2 variables; using the variables as the embedding ^10^. The simplification in the model and resulting approximation may produce suboptimal results especially in ‘local’ microstructure of the data, but the strict separation brings more beneficial properties: The necessary generalization prevents overfitting and thus improves the applicability of the model to newly incoming data. Performance of the algorithm is usually improved, because fitting of the data to the constant trained model can be trivially accelerated by parallelization.

In EmbedSOM, the model consists of the pairs of the corresponding high- and low-dimensional landmarks ( *L* and *l*) created by any suitable algorithm (including SOMs, autoencoders, and any of the optimization-based dimensionality reduction described above); fitting of the data into the model is then performed by minimizing the total projection error for each data point separately.

The geometrical interpretation of EmbedSOM bears similarity to linear dimensionality reduction methods — the projection is locally linear, and the non-linearity is caused only by the non-linear weighting of landmark influences (scores *S* in Algorithm 1). With SOMs, the result can thus be viewed as many local PCA projections smoothly stitched together. For extreme parameter settings (4 landmarks generated by a 2×2 SOM, *k* = | *L*| = 4, *a* = *b* = 0 and *m* = ∞), EmbedSOM produces results almost identical to PCA.

### Generalized landmarks and GQTSOMs

While the SOMs are a great method to generate landmarks *L* and *l* that carry various beneficial properties that simplify human interpretation of the result (notably the regularity of *l*), other methods are admissible as well, as long as they can cover the input space sufficiently by *L* and generate the corresponding landmarks *l* in the low-dimensional output space so that the topology is similar to *L*.

For example, the embedding process can be simplified to a great extent by completely removing SOMs: Instead of constructing *L* in a complicated way so that it reflects the input space topology, we can take only a small random sample of input points as the landmarks, and use a general DR method to find its topology and arrange landmarks *l* in a matching way, as shown in Figure 1 on an example with t-SNE. While this is often sufficient, for the purposes of embedding it is more beneficial to find a smaller set of landmarks that provide better description of the various features in the input space than the random sampling.

Many variants of the SOM algorithm have been created to optimize this metric: For example, the Growing SOMs (GSOMs) by Dittenbach *et al.* ^11^, start with a simple 2×2 SOM grid, and dynamically add new SOM grid vertices at the SOM perimeter only if it is necessary to keep the total quantization error low. A hierarchical variant of GSOM called GHSOM introduced by Rauber *et al.* ^4^ aims to improve the description of small details in the input data space that were not described sufficiently by GSOMs. Depending on the heuristic, the vertices of GHSOM grid are converted to small independent versions of GHSOMs, which map the corresponding local parts of the input space; this continues recursively to create a layered structure of SOMs that describe increasingly fine and subtle details in the data.

**GQTSOMs** Although the GHSOMs improve the classification of small-scale features in the datasets, the hypertree structure complicates their use as landmarks for planar visualization with EmbedSOM. We propose the Growing QuadTree-structured SOMs (GQTSOMs) to alleviate this problem: The GQTSOMs grow by recursively splitting the nodes to form a hypertree, but unlike GHSOMs the hypertree shape is restricted to a quadtree, which possesses straightforward interpretation as a 2-dimensional structure ^12^.

The nodes in GQTSOMs are identified by their position and depth in the quadtree, represented as an integer triple ( *L*, *x*, *y*). The corresponding 2-dimensional coordinates are obtained as (2 *x* + 1, 2 *y* + 1) ⋅ 2 ^− *L*^. Initial nodes in training occupy positions on a regular grid with *L* = 0. Upon growing, a node ( *L*, *x*, *y*) is split into 4 nodes identified as ( *L* +1, 2 *x*, 2 *y*), ( *L* +1, 2 *x* +1, 2 *y*), ( *L* +1, 2 *x* +1, 2 *y* +1), and ( *L* +1, 2 *x*, 2 *y* +1). Figure 1 shows an example of 3-level GQTSOM in a 2-dimensional space, where the initial 3×3 SOM grew 7 times to produce 30 landmarks.

GQTSOM training proceeds by batches as in the usual batch SOM training. After each epoch, several nodes with greatest position change in the input data space are split, so that the total number of nodes grows linearly during the whole training. Initial positions for the new nodes are interpolated from the topological SOM neighborhood, using the same neighborhood function as for training the SOM (e.g. a Gaussian). To avoid overcrowding of the map by small nodes and promote their specialization to fine details, the nodes are penalized by a factor of *L* ^−1^ in the growing heuristic, and by a factor of 4 ^*L*^ applied to their neighborhood volume in both input space and SOM space.

### Implementation

The current version of EmbedSOM is available as R package EmbedSOM from http://github.com/exaexa/EmbedSOM, together with the customized versions of SOM and GQTSOM algorithms. The implementations are conducted in C++ independent of the R wrapping, and can be reused in other environments. The integration into R serves mostly as a bridge to the large number of cytometry-oriented packages in the ecosystem.

Low-level implementation has provided several ways to improve the performance of the algorithms when compared to the original implementation: For example, cache-efficient version of the SOM training has improved the performance by up to 15× on SOMs larger than 40×40; SIMD-based acceleration of the vector operations by up to 4×, and parallelization of the batch SOM training and embedding by a factor roughly equivalent to the number of used CPUs.

Overall, the computation time required for typical datasets was reduced by a factor greater than 10× on commonly available hardware, and often more than 30× in case of processing complicated datasets using very large SOMs on highly parallel hardware.

### Operation

For single-cell analysis, EmbedSOM is best used from R environment; the package can be downloaded from GitHub using R command devtools::install_github('exaexa/EmbedSOM'). The package installation will automatically compile the code that uses the SIMD capabilities if they are enabled on the target platform.

Generally, the SOM and embedding process can be executed on any real matrix with individual data points in rows, and parameters in columns. This expectation is consistent with many other DR or clustering packages, including FlowSOM, Rtsne and umap. For example, a user may obtain an embedding of the Iris dataset as such:


library(EmbedSOM)                                                
d <- iris[,1:4]                                                    
map <- SOM(d)                                                     
e <- EmbedSOM(map=map, data=d)                                     


In the code, the landmarks are first created using a SOM and saved in the map, which is then passed to the EmbedSOM function that produces the final 2-column matrix e with embedded coordinates. These can be plotted e.g. using the standard plot function.

On data larger than Iris dataset, GQTSOMs may be used to generate the landmarks and a map usable with EmbedSOM function in a similar way:


map <- GQTSOM(d, target_codes=500, parallel=T)                   


Here, target_codes chooses the desired final number of the landmarks in the fully grown SOM, and parameter parallel=T allows the computation to use multiple available CPUs. Functions SOM and EmbedSOM support parallelization as well, using the same parameter.

Other DR methods may create the landmarks. For example, the following code generates a map object with 500 landmarks projected with t-SNE, suitable for t-SNE-directed embedding:


library(Rtsne)       
landmark_idx <- sample(nrow(d) , 500)         
map <- list(codes=d[landmark_idx,], grid=Rtsne(d[landmark_idx,])$Y)


The parameters of the SOM, GQTSOM and EmbedSOM functions are extensively documented in the supplied R manual pages.

## Use cases

**Figure 2.**
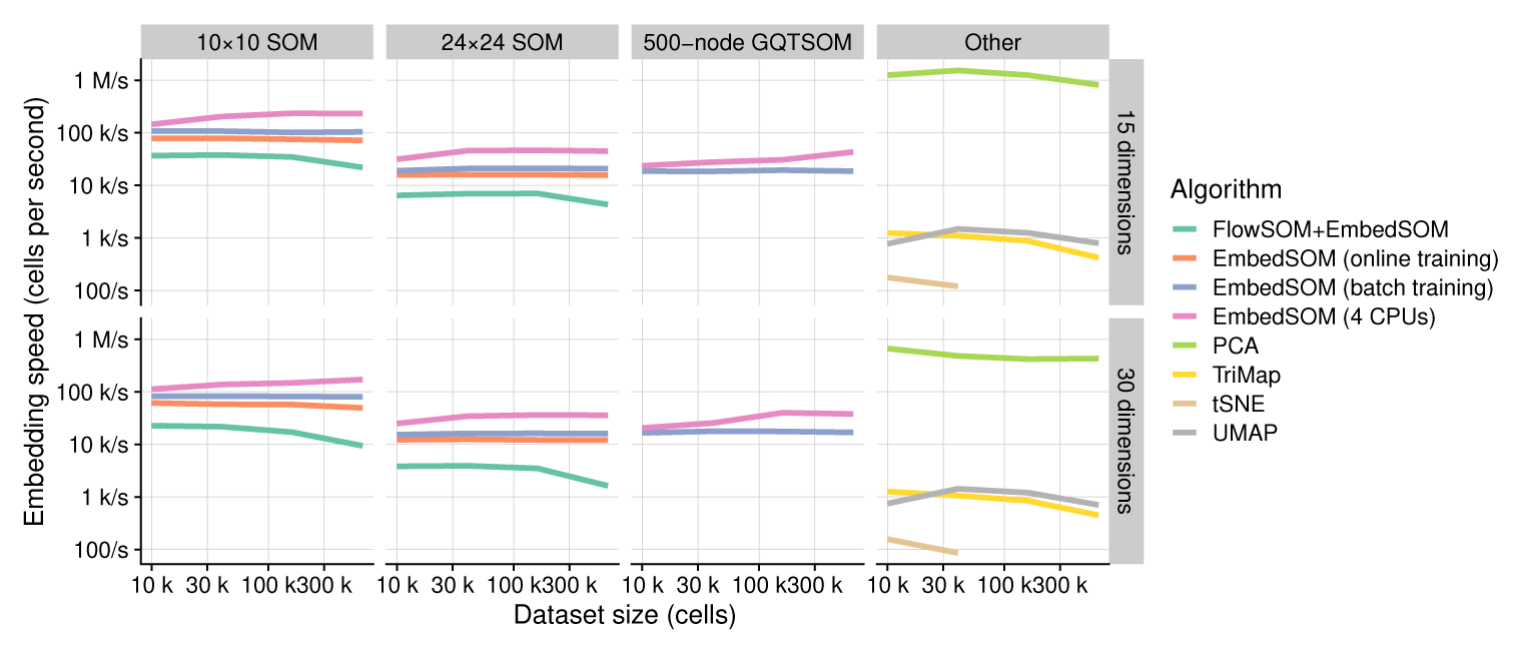
Performance of EmbedSOM variants compared with other dimensionality reduction methods. The speed is represented in cells per second. EmbedSOM-based algorithms show almost perfect linear scaling with growing dataset size, and even minor speed improvements when sufficient data is available for saturating the parallel computation. As expected from their asymptotic complexities, performance of UMAP, TriMap and t-SNE decreased with additional data. t-SNE was not executed on datasets larger than 50 thousand cells because of time constrains.

The primary purpose of EmbedSOM is to produce quickly available and highly comprehensible data visualization in situations where processing speed and efficiency is critical. The embedding time of the demonstration datasets was measured on an AMD Ryzen 7 2700U CPU with 16GB of RAM running Debian Linux (Bullseye), R version 3.6.2 compiled with gcc version 9.3; the timing is reported in the corresponding figures as *t*, together with number of cells ( *n*) and landmarks (| *L*|). Comparison of embedding speed with other popular dimensionality-reduction methods can be seen in Figure 2. As the main result, the measurements show that a high-quality visualization of a data file from a common experiment (around 300 thousand cells) can be obtained in less than 10 seconds using common office hardware.

Here, we demonstrate EmbedSOM functionality on two use-cases: First, using the described variants of landmark-generating functions, we reproduced the visualizations by Becht *et al*. ^6^ of a dataset that maps specific trafficking and cytokine signatures of human T cells across tissues, created by Wong *et al*. ^13^. Second, we visualized a human gastrointestinal disorders dataset by van Unen *et al*. ^14^ using GQTSOMs, showing that EmbedSOM provides a viable alternative to the semi-interactive analysis of rare cell types using the HSNE algorithm ^15^.

### Alternative landmark-generating methods improve visualization

**Figure 3.**
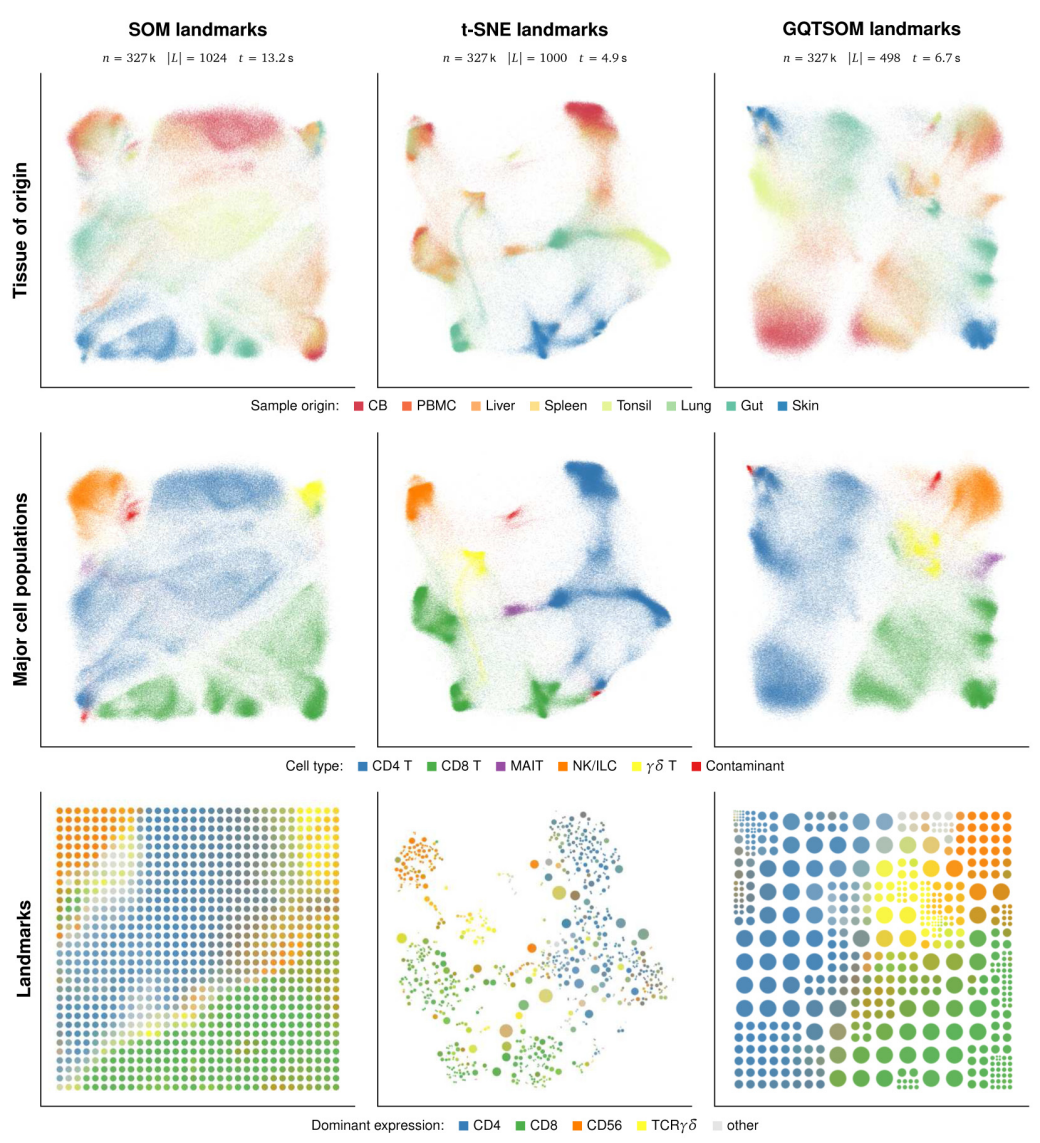
Comparison of EmbedSOM visualizations of the Wong dataset using different landmarks. Top row: cells embedded using 3 different landmark-generating methods, colored by the tissue of sample origin. Middle row: The same embedding colored by major cell types. The colors used for annotation are purposefully reproduced from the article of Becht *et al.* ^6^ to simplify comparison. Bottom row: visualizations of the low-dimensional landmark images, colored by their corresponding marker expressions.

To visualize the Wong dataset, we have run EmbedSOM algorithm with the SOM landmarks, t-SNE generated landmarks, and GQTSOM-generated landmarks. As seen in Figure 2, the original EmbedSOM implementation has managed to separate and visualize both the different cell types and their layout according to source organ. However, the result may seem unsatisfactory due to overcrowding and loss of both detail and global layout, especially when compared to UMAP visualizations of the same dataset [ 6, Figure 1a,b]. Despite the overcrowding, it is still possible to identify clusters of CD69 ^+^CD103 ^+^ Trms (resident-memory T cells) in all organs except cord blood, and naive (CD69 ^+^CD45RA ^+^), central memory (CCR7 ^+^CD62L ^+^) and effector memory T cells (CD45RA ^–^ CD45RO ^+^CCR7 ^–^CD62L ^–^) within both CD4 and CD8 T cell types; this is in agreement with findings of van Unen *et al.* [ 14, Figure 3a,b]. Plots of all marker expressions are available as *Extended data*.

Improved methods of landmark positioning have successfully alleviated both overcrowding and layout problems. In particular, the layout of MAIT (mucosal-associated invariant T) and *γδ* T cells in the embedding with t-SNE-generated landmarks reflects the expected properties of cell populations, and the individual population clusters are clearly separated by low-density areas with intermediate cell states and noise. The usefulness of the smoothness property can be observed on the cluster of *γδ* T cells, where EmbedSOM shows a similarity of the gut-originating part of *γδ* T cells to both gut-originating CD8 ^+^ T cells and other types of *γδ* T cells, even though this is neglected by the underlying t-SNE. In comparison, this connection is preserved by all tested types of SOMs, but neglected by both plain t-SNE and UMAP, which show the population separated to 3 resp. 2 separate clusters [ 6, Figure 1a]. The embedding based on GQTSOM landmarks has provided similar global layout of the output as the one with t-SNE landmarks, additionally capturing the continuity of *γδ* T cell cluster and its similarity to MAIT and NK cells, and providing separation of individual clusters differentiated by tissue of origin comparable to that of UMAP. Compared to the SOM used with the original EmbedSOM approach, GQTSOM generates a smaller amount of more precise landmarks, which resulted in significant computation speed increase (around 50%) and better description of the small and rare cell populations by landmarks. In particular, the small subpopulations of *γδ* T cells were assigned roughly twice the number of landmarks by GQTSOM than by the standard SOM, which resulted in spatially correct separation of the cell subtypes in the embedding.

### GQTSOM landmarks improve display of rare cell types

We showcase the ability of GQTSOM landmark generation method to capture and display various rare cell types using a dataset by van Unen *et al.* ^14^ The dataset was created as such: A total of 5.2 million single cells were collected from duodenum biopsies, rectum biopsies, perianal fistulas, and PBMC from patients undergoing various gastrointestinal disorders and healthy individuals (as controls). The gastrointestinal disorders included celiac disease (CeD), refractory celiac disease type-II (RCDII), enteropathy associated T-cell lymphoma type II (EATLII), and Crohn’s disease. Cells were stained using 32 metal conjugated monoclonal antibodies to identify cells within the innate and adaptive immune system. This dataset was later reanalyzed by van Unen *et al.* ^15^ using a hierarchical version of t-SNE algorithm called HSNE, showing that the hierarchical dissection of the data was able to identify several rare cell types within the innate lymphoid cell (ILC) compartment.

**Figure 4.**
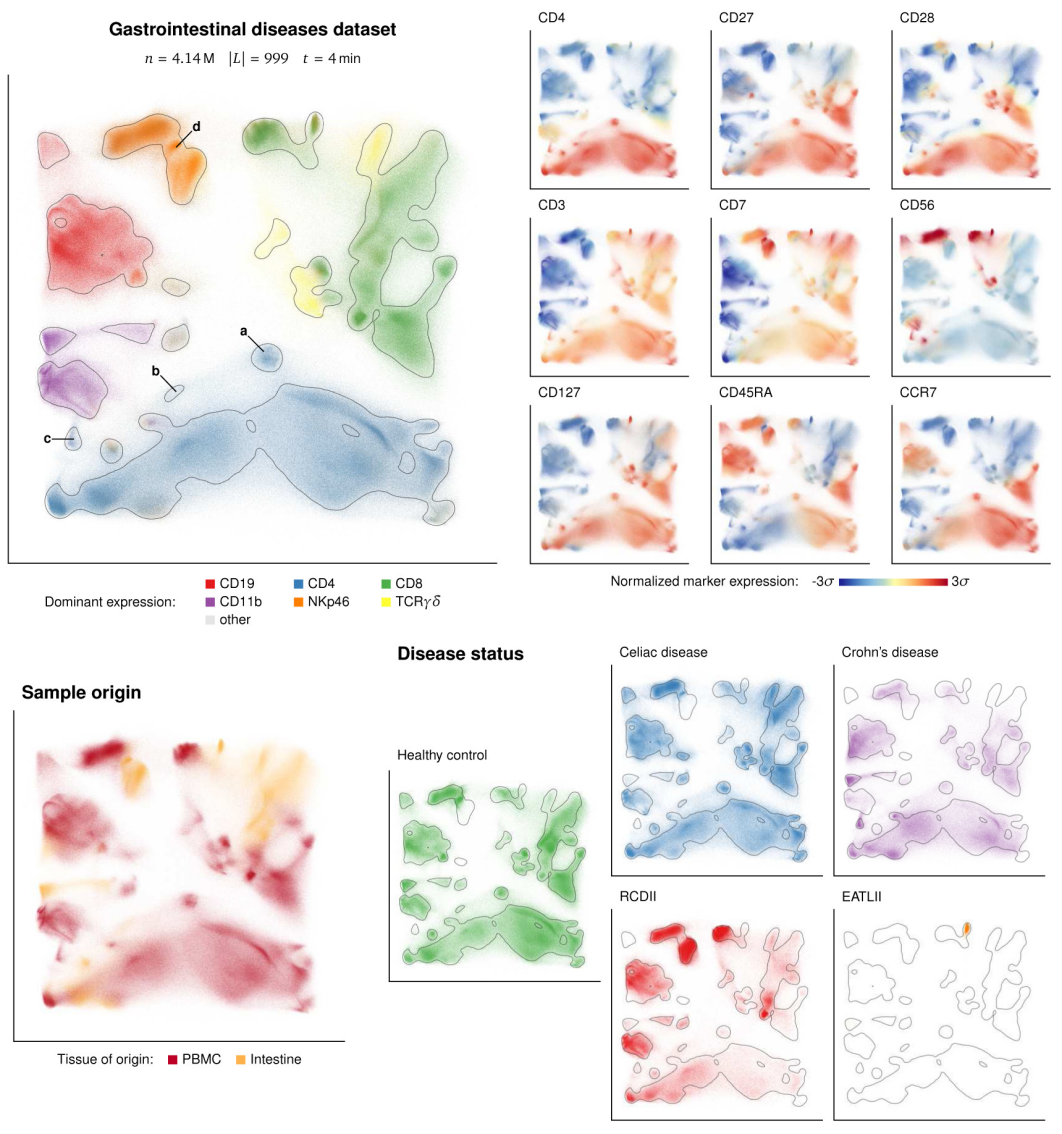
Display of clusters of rare cell types in GQTSOM-based embedding. Top left: Overview of the cleaned and embedded Unen dataset, colored by expression of main cell lineage markers. The contour based on Gaussian difference is added for easier identification of changes in cell density. Labels mark the rare cell types identified by van Unen *et al*. ^15^, Belkina *et al*. ^16^: (a) CD4 ^+^CD28 ^–^CCR7 ^+^, (b) CD4 ^+^CD28 ^–^CCR7 ^–^CD56 ^–^, (c) CD4 ^+^CD28 ^–^CCR7 ^–^CD56 ^+^, and (d) CD7 ^+^CD3 ^–^CD127 ^–^CD45RA ^–^CD56 ^partial^. Top right: Expressions of separate markers used for the identification. Bottom: Cells color-coded by sample origin (left) and separated by disease status of the patient (right).

For the purpose of demonstration, we preprocessed the same dataset by removing debris, doublets and dead cells based on simple thresholds on the DNA, Event length and Viability parameters. The 32 antibody markers of the 4.14 million cleaned cells were then transformed by hyperbolic arcsine and used to train the GQTSOM and produce an embedding. The result in Figure 4 allows easy observation of both the ILC compartment and the CD4 ^+^ T cell subset, corresponding to the observations produced by second-level HSNE [ 15, Figures 3 and 5]. Additionally, the embedding shows presence of many clusters from lower levels of the hierarchical dissection: In the figure, it is possible to identify clusters of CD4 ^+^CD28 ^–^CCR7 ^–^CD56 ^–^ and CD4 ^+^CD28 ^–^CCR7 ^–^CD56 ^+^ rare cell types within the CD4 ^+^ compartment, and of the CD127 ^–^CD45RA ^–^CD56 ^partial^ cluster within the ILC (CD7 ^+^CD3 ^–^) compartment. These clusters were identified by HSNE at 4 ^th^ resp. 3 ^rd^ levels of dissection [ 15, Figures 5b and 3c]. Recently, Belkina *et al.* ^16^ showed that the opt-SNE algorithm can additionally identify CD4 ^+^CD28 ^–^CCR7 ^+^CD56 ^–^ rare cell type, which is also clearly separated by the GQTSOM-based embedding, using much less computational resources than optSNE.

The plot of cells separated by disease status in Figure 4 confirms the observation that the rare CD4 ^+^CD28 ^–^CD56 ^+^ phenotype is enriched in the samples from patients with Crohn’s disease. Moreover, the plot gives a useful overview for identifying cell types specific for the other diseases, showing two specific and one enriched cluster for RCDII, a single specific cluster of CD8 ^+^CD56 ^+^CD127 ^+^c-KIT ^+^ cells for EATLII, and one specific and some enriched cell types in patients with CeD.

## Summary

We have presented an improved and generalized version of EmbedSOM, supported by the new model of quadtree-structured growing self-organizing maps. The functionality of the new algorithm was demonstrated on data and analyses from recent studies, showing that the new combination provides superior embedding speed and good rendering of various cell types, including tissue-specific and rare phenotypes.

## Software availability

- Source code available from: https://github.com/exaexa/EmbedSOM
- Archived source code available from: https://doi.org/10.5281/zenodo.3568980
- Software license: GNU GPLv3

## Data availability

The used datasets are freely available from FlowRepository.org under accession IDs:

- FR-FCM-ZZTM (Wong dataset; the data was preprocessed exactly as described by Becht *et al.* ^6^)
- FR-FCM-ZYRM (Unen dataset)

Supplementary code and visualizations of the embedded datasets are available on FigShare, under DOI 10.6084/m9.figshare.11328035
