# Supplementary material for: Generalized EmbedSOM on quadtree-structured self-organizing maps
Source: F1000Res. 2020 May 19;8:2120. Originally published 2019 Dec 18. [Version 2] doi: 10.12688/f1000research.21642.2 (PMC7255855; doi:10.12688/f1000research.21642.2)
Supplement: Peer review history

# Peer review: Reviewer response for version 2

**DOI:** 10.5256/f1000research.26529.r63640

**Published:** 2020-05-26

**Authors:** Nikolay Oskolkov

**Affiliations:** 1 Department of Biology, National Bioinformatics Infrastructure Sweden, Science for Life Laboratory, Lund University, Lund, Sweden

The concerns previously raised by me have been properly addressed and I believe the manuscript is ready for publication

Are the conclusions about the tool and its performance adequately supported by the findings presented in the article?

Yes

Is the rationale for developing the new software tool clearly explained?

Partly

Is the description of the software tool technically sound?

Yes

Are sufficient details of the code, methods and analysis (if applicable) provided to allow replication of the software development and its use by others?

Partly

Is sufficient information provided to allow interpretation of the expected output datasets and any results generated using the tool?

Yes

Reviewer Expertise:

Computational Biology, Bioinformatics, Mathematical Statistics and Machine Learning

I confirm that I have read this submission and believe that I have an appropriate level of expertise to confirm that it is of an acceptable scientific standard.

# Peer review: Reviewer response for version 1

**DOI:** 10.5256/f1000research.23857.r59220

**Published:** 2020-02-25

**Authors:** Nikolay Oskolkov

**Affiliations:** 1 Department of Biology, National Bioinformatics Infrastructure Sweden, Science for Life Laboratory, Lund University, Lund, Sweden

The authors present an improved version of EmbedSOM that was optimized for speed to address extra large single cell data sets. In addition, a novel way of growing SOM based on quad-tree was proposed.

 Dimension reduction of large single cell RNAseq and flow cytometry data sets is very challenging due to the crowding problem and algorithm scalability issues. Therefore, developing methods alternative to the current golden standards such as PCA, tSNE and UMAP is of high importance. More specifically, tSNE and UMAP are capable of preserving only local structure while PCA keeps the global structure information. However, no method is currently available that can preserve both local and global structure.

 Self-Organizing Maps (SOMs) and the modified EmbedSOM that are discussed in the manuscript represent an interesting and promising algorithm in this respect. However, I would like to raise a few questions and concerns to be addressed by the authors.

 First, based on the cost function mentioned on the page 3, the algorithm seems to resemble MDS / PCA type of dimension reduction. Therefore, I would like to see a comparison of EmbedSOM with MDS / PCA. If a connection between the gamma-delta T cells and CD8 T cells was not captured by tSNE and UMAP as it is mentioned on the page 8, probably due to the lack of global structure preservation by tSNE and UMAP, was this connection captured by MDS / PCA?

 Second, what would be the benefit of using EmbedSOM compared to PCA / MDS, tSNE and UMAP? Do we discover any new biology using EmbedSOM that is not captured by PCA / tSNE / UMAP? Do we benefit from the computational speed of EmbedSOM compared to PCA / tSNE / UMAP? If so, is it really faster (and how much faster) than PCA? I would like to see a clear formulation of the role of the EmbedSOM among other dimension reduction methods.

 Third, I was really impressed by the Figure 1 and how well GQTSOM-based embedding was able to reconstruct the original 3D S-shaped non-linear manifold. To my experience, tSNE / UMAP and especially PCA / MDS would have difficulty reconstructing the 3D S-shaped manifold as 2D embeddings. I have not found any links to the codes for reproducing this embedding and would be very curious to see whether GQTMSOM / EmbedSOM is really capable of capturing the internal 2D structure of the 3D S-shaped non-linear manifold.

Are the conclusions about the tool and its performance adequately supported by the findings presented in the article?

Yes

Is the rationale for developing the new software tool clearly explained?

Partly

Is the description of the software tool technically sound?

Yes

Are sufficient details of the code, methods and analysis (if applicable) provided to allow replication of the software development and its use by others?

Partly

Is sufficient information provided to allow interpretation of the expected output datasets and any results generated using the tool?

Yes

Reviewer Expertise:

Computational Biology, Bioinformatics, Mathematical Statistics and Machine Learning

I confirm that I have read this submission and believe that I have an appropriate level of expertise to confirm that it is of an acceptable scientific standard, however I have significant reservations, as outlined above.

# Response

**Published:** 2020-04-16

**Authors:** Miroslav Kratochvíl

**Affiliations:** Department of software engineering, Faculty of Mathematics and Physics, Charles University, Prague, Czech Republic

Thank you for the review and for pointing out the deficiencies. We believe that the raised points should be addressed -- especially regarding the comparison of EmbedSOM with other dimensionality reduction methods (adding some helpful hints for the readers to decide whether they should use EmbedSOM) and the code that produces the Figure 1 and processes the other dataset (thus improving availability and reproducibility). We plan to submit the extended version of the article in several days.

# Peer review: Reviewer response for version 1

**DOI:** 10.5256/f1000research.23857.r57989

**Published:** 2020-02-11

**Authors:** Evan Newell, Hugh MacMilan

**Affiliations:** 1 Fred Hutchinson Cancer Research Center, Seattle, WA, USA

The authors generalize their EmbedSOM approach to examine two additional ways of selecting the respective sets of landmarks in the high- and low-dimensional spaces, beyond the standard SOM, to address. Example data analyses are appreciated.

 That selection is the first stage in the embedSOM approach. The second stage is the actual embedding enrichment process. 

 The authors explain: "EmbedSOM projection can be viewed as an embedding enrichment method: From a set of landmarks in the high-dimensional space and a set of corresponding landmarks in the low-dimensional space, it produces a smooth function that maps all points from the higher-dimensional space to the low-dimensional space and preserves the relative neighborhoods of the landmarks."

Testing:

- We followed the paper’s guidance on some in-house fcs files and had success with embedSOM and the  GQTSOM function.

Some naming confusion:

- By "generalized EmbedSOM” the authors refer to using different ways of generating landmarks, other than the original (self-organizing map) SOM approach. 
- It seems preferable to drop the “SOM” rather than refer to these variants as "generalized EmbedSOM” methods.  The authors might use the more general notion of landmarks, rather than SOM. As they note, the random-sampling, followed by tSNE, version of “generalized EmbedSOM” doesn’t use SOMs at all. 

Re “compacting noise"

- The first reference of the manuscript includes some background on differences between the “generalized EmbedSOM” approach and what the authors call “plain tSNE and UMAP,” and attributes these differences to the respective designs of the algorithms. 
- In that background paper, the authors explain: “neither UMAP nor tSNE aim to preserve local linearity of the transformation, which allows them to take apart the clusters with noisy data and attach the residual noise to nearest clusters.”  
- They concluded in that paper: “Compacting the residual or unexplained noise is desirable for providing a clean display of the data for publication. On the contrary, almost-immediate availability of all information about very large datasets, including the (often informative) noise, is more important for producing comprehensive graphics for high-throughput analysis." 
- This paper marks an attempt to explore those differences, and the apparent trade-offs, in more detail, so it would benefit from discussing these tradeoffs in the context of the algorithm designs.
- The authors noted in the first reference, "While the observed cluster separation may be desirable if the embedding is expected to approximate the population boundaries, it may be inappropriate if the population environment is relevant for analysis."

GQTSOMs:

- The manuscript introduces a new landmark-generating algorithm that simplifies a hierarchical variant of an adaptive SOM approach, namely, growing quad tree SOMS (GQTSOMs) as a simplified growing hierarchical SOM (GHSOM), which is in turn a variant of growing SOMs (GSOMs). 
- The aim is to identify and incorporate features in the input space more efficiently than random sampling, by using a "layered structure of SOMs". This is a natural thing to do to improve on SOM.
- On page 8 the authors report that GQTSOM leads to using a “smaller amount of more precise landmarks” and thus faster computation, and appears to be a nice contribution.

Are the conclusions about the tool and its performance adequately supported by the findings presented in the article?

Yes

Is the rationale for developing the new software tool clearly explained?

Yes

Is the description of the software tool technically sound?

Yes

Are sufficient details of the code, methods and analysis (if applicable) provided to allow replication of the software development and its use by others?

Yes

Is sufficient information provided to allow interpretation of the expected output datasets and any results generated using the tool?

Yes

Reviewer Expertise:

Cellular immunology

We confirm that we have read this submission and believe that we have an appropriate level of expertise to confirm that it is of an acceptable scientific standard.

# Response

**Published:** 2020-02-22

**Authors:** Miroslav Kratochvíl

**Affiliations:** Department of software engineering, Faculty of Mathematics and Physics, Charles University, Prague, Czech Republic

Thank you for the review and comments. We will wait for the additional reviews and address some of your suggestions in the second version of the manuscript.

Regarding the name of EmbedSOM, we are aware of the issue with textual "specialization" to SOMs which ignores the modifiable parts of the workflow, but since the package is already published over a year and we had not been able to invent a strictly better name so far, we expect that the name will stay. We will gratefully accept suggestions (also from readers) for a reasonably short name that sufficiently characterizes the projection procedure.
